# Key characteristics of health research systems for health emergencies preparedness and response: a scoping review

**DOI:** 10.1136/bmjph-2025-004023

**Published:** 2026-11-02

**Authors:** Nicolas Pulik, Adebisi Adenipekun, Sassy Molyneux, Alice Norton

**Affiliations:** 1 Pandemic Sciences Institute, University of Oxford, Oxford, UK; 2 Centre for Tropical Medicine and Global Health, University of Oxford, Oxford, UK

**Keywords:** Public Health, Disease Outbreaks, Epidemics, Scoping Review

## Abstract

**Objectives:**

Research systems have rapidly evolved to support infectious disease outbreak response but major gaps remain, particularly in governance and financing. Given limited funding, strengthening research systems requires identifying evidence-based priorities. However, literature on how health research systems function during emergencies is limited. This review maps the key characteristics of health research systems for emergency preparedness and response, and the indicators used to assess their capacity.

**Design:**

The scoping review was conducted according to Joanna Briggs Institute guidelines and the Arksey and O’Malley framework and reported according to the Preferred Reporting Items for Systematic Reviews and Meta-Analyses extension for Scoping Reviews guidelines.

**Data sources:**

We searched Embase, Medline, Global Health, Scopus, the WHO Global Index Medicus and Google Scholar on 24 June 2025. We included 36 resources that discussed at least one element of the health research systems conceptual framework (Jones *et al*) and were linked to health emergencies caused by high-consequence infectious pathogens.

**Data extraction and synthesis:**

Data were extracted using a refined data extraction sheet and analysed through inductive and deductive thematic analysis based on the Jones framework for health research systems. Pearson correlation analysis was used to assess relationships between health research system characteristics.

**Results:**

The review reveals the complex interplay within health research systems. Prominent themes included ‘Creating and sustaining resources’, ‘Producing and using research’ and ‘Financing’, linked to emerging themes such as ‘Pace’, ‘Flexibility’ or ‘Integration’. No indicators were specifically designed to assess outbreak preparedness or responsiveness, and relevant measures were loosely defined.

**Conclusion:**

This study identifies priorities for funders and policymakers to strengthen health research systems. Sustainable, long-term investment across all system dimensions is essential for effective preparedness and response. There is a need for further evidence and evaluation around health research systems and the role they fulfil during health emergencies.

**WHAT IS ALREADY KNOWN ON THIS TOPIC**

**WHAT THIS STUDY ADDS**

- This study highlights characteristics of health research systems considered important during health emergencies which had not been captured systematically before. It provides a conceptual framework for health emergencies and a qualitative systemic analysis of the interrelation between the various components for health research systems during emergencies.

**HOW THIS STUDY MIGHT AFFECT RESEARCH, PRACTICE OR POLICY**

- This study aims to help decision makers streamline investments into preparedness and response. It makes a timely case for investing in health research systems capacity strengthening, given many governments are currently considering disinvesting.

## Introduction

### Health research systems

 Over the last two decades, health research systems have been confronted with recurring health crises such as the H1N1 pandemic, the West Africa Ebola epidemic and the COVID-19 pandemic. While health research systems have achieved unexpected results in record times during the SARS-CoV-2 pandemic, there remain inefficiencies in the way these systems function during health emergencies, notably in financial resources allocation or emergency coordination, and uncertainties about the necessary characteristics to improve resilience.^1–4^ Therefore, this study aims to identify and map the components and evaluative measures of health research systems considered important to health emergency preparedness and response.

Health research systems can be defined as ‘the people, institutions, and activities whose primary purpose in relation to research is to generate high-quality knowledge that can be used to promote, restore, and/or maintain the health status of populations; it should include the mechanisms adopted to encourage the utilisation of research’.^5^ These systems are responsible for the production and promotion of knowledge which ultimately aims to improve health. Health research systems have been conceptualised since 2003^5^; however, for the purpose of this review, we will use a more recent framework developed in 2021 by Jones and coauthors^6^ (figure 1). This framework builds on the earlier framework but further integrates supporting elements and ongoing processes of health research systems which makes it more comprehensive and allows considering the complex and dynamic relationships between the various characteristics of these systems.^6^ It is made of functions, components, ongoing processes and supporting elements (table 1).

**Figure 1:**
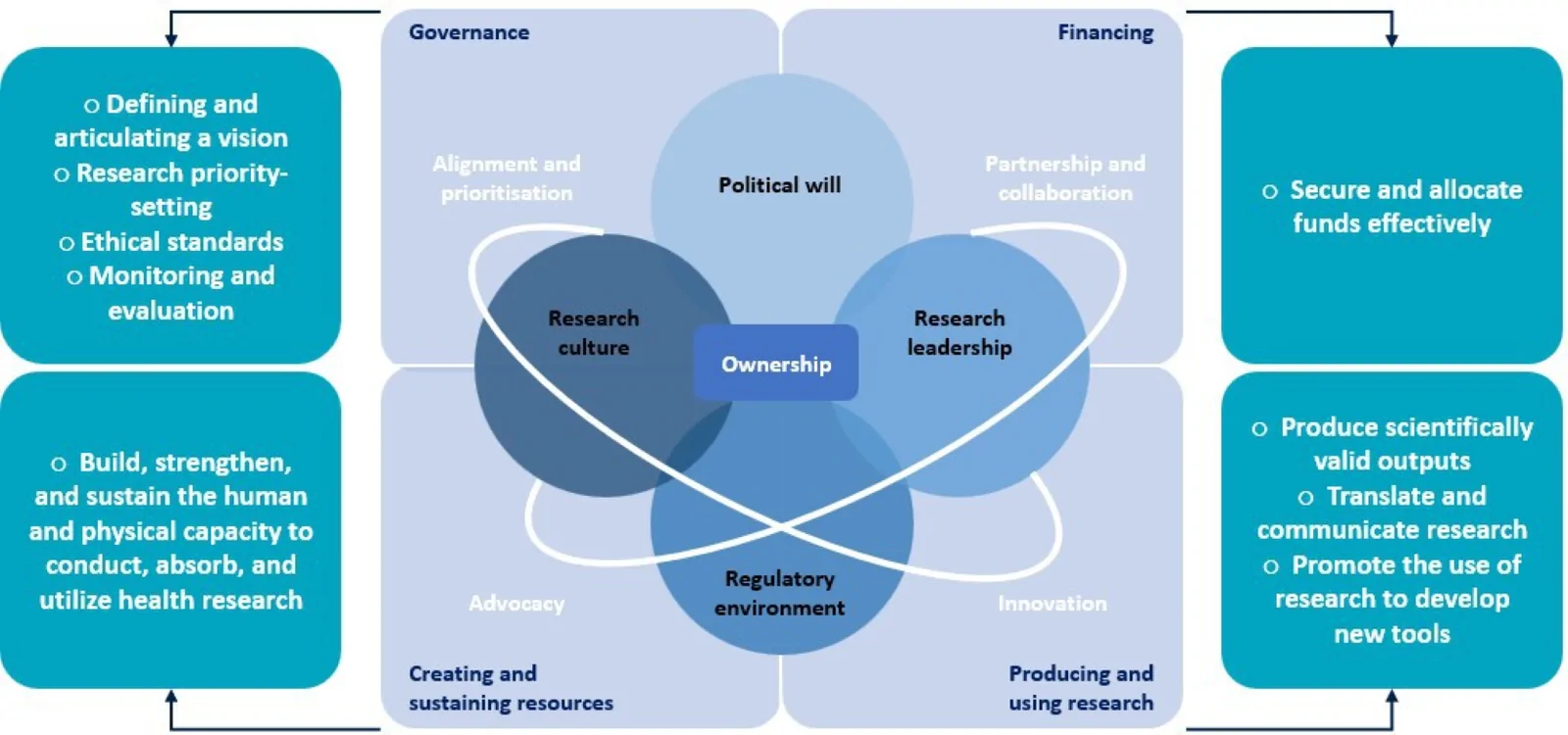
Graphical representation of the conceptual framework used for the scoping review. The figure is reproduced from Jones *et al*^6^ and augmented.

**Table 1:** Definitions of the main concepts used in the Jones conceptual framework used in this scoping review

Concepts	Examples	Definitions
Functions/pillars*	Governance, Financing, Creating and sustaining research, Producing and using research	These are the main structural elements that constitute the basis for a functioning health research system.
Components*	Define and articulate vision for a national health research system, Produce scientifically valid research outputs, …	These are the operational pieces of the functions of health research systems.
Supporting elements†	Regulatory environments, Political will, Research leadership, Research culture	‘Supporting elements(…)serve a relational role to link up different parts of the system’.
Ongoing processes†	Collaboration, Advocacy, Alignment and prioritisation and Innovation	*‘*Ongoing processes(…)serve as practices and methods used by actors in the (national health research system) to support (its) functioning and (its) improvement*’*.

* The definitions for functions and components are adapted from Pang *et al*.

† The definitions for supporting elements and ongoing processes are reproduced from Jones *et al*.

### Health research systems in the context of health emergencies

Health emergencies can be defined as outbreaks caused by high-consequence infectious diseases. These can be defined as ‘infectious disease pathogens which cause diseases in humans with the potential to cause outbreaks associated with devastating morbidity and mortality*’*.^7^ The list of high-consequence pathogens that we used as a reference can be found in online supplemental appendix 1.

During health emergencies, health research systems are central to provide effective diagnostics, treatments, vaccines as well as public health and social measures to prevent the spread of diseases. They are the cornerstone of effective response to high-consequence infectious pathogens emergencies. Yet, very often they are absent from response plans. For instance, the International Health Regulation (IHR) update, published in 2005, did not mention the word research.^8^ The update to the IHR, published in 2024, has reviewed article 13 to include research in two instances to encourage ‘equitable access to relevant health products’.^9^ As a consequence, their effectiveness has been questioned during recent outbreaks. While a number of initiatives have flourished to strengthen health research systems, such as the WHO R&D Blueprint for Epidemics^10^ and the Coalition for Epidemic Preparedness Innovations,^11^ the COVID-19 pandemic has highlighted remaining gaps and issues.^1–4^ In April 2025, the Intergovernmental Negotiating Body has reached a draft Pandemic Agreement in which Article 9 is dedicated to research and development and recognises the need to ‘build, strengthen and sustain geographically diverse capacities and institutions for research and development […] and shall promote research collaboration, access to research and rapid sharing of research information and results, especially during public health emergencies of international concern, including pandemic emergencies’.^12^

Health research systems have not been studied systematically and much of the literature on health research systems and health emergencies has only emerged since 2020. A study, published in 2022, explored the success and challenges of health research systems during the COVID-19 pandemic in seven selected countries and showed that these systems would benefit from ‘greater coordination and integration […] both of which can be achieved via a comprehensive health research strategy’.^1^ Another study, published in 2025, demonstrated potential options for health research systems improvement towards better preparedness based on an environmental scan of relevant literature in high-income countries.^13^ Another recent commentary gave an overview of health research systems leads for reinforcement.^14^ Given the current atmosphere of global health funding contraction and undermining of health research systems, it is essential to show how these systems contribute to improving health and protecting populations globally.

The primary objective of this scoping review is to identify and map the elements of health research systems that are considered, in the literature, as key to health emergencies preparedness and response. The secondary objective of this review is to identify and map indicators or tools to monitor the effectiveness of health research systems during health emergencies.

## Methods

### Protocol and registration

The scoping review was conducted in accordance with the Joanna Briggs Institute Manual for Scoping Reviews^15^ and the Arksey and O’Malley framework.^16^ The reporting followed the Preferred Reporting Items for Systematic Reviews and Meta-Analyses extension for Scoping Reviews guidelines (PRISMA-ScR).^17^ This research did not involve patients or the public in its design, conduct, reporting or dissemination plans.

A protocol for this scoping review was registered on the Open Science Framework.^18^ A few amendments have been installed. First, we used the concept of health emergencies—instead of pandemics—to broaden the research scope. This also ensures its relevance and usefulness, since pandemics are rare, but health emergencies relatively frequent. This had no impact on the search strategy which was designed to be comprehensive. We added a list of high-consequence infectious diseases to facilitate screening (online supplemental appendix 1).

### Eligibility criteria

Resources that did not have a link to at least one element of the health research systems conceptual framework, as well as a link to health emergencies caused by high-consequence infectious pathogens (as listed in online supplemental appendix 1) were excluded. Books were excluded for practical reasons. We excluded resources that were not in English. We did not use any time or geographical boundaries.

### Information sources

We searched Embase (Ovid), Medline (Ovid), Global Health (Ovid), Scopus and the WHO Global Index Medicus. We also searched the first 10 pages of Google Scholar.

### Search strategy

The search strategy was designed to be as comprehensive as possible, capturing all relevant resources on health research systems, given the limited field of health research systems in health emergency preparedness and response. The search was piloted on Medline and Embase and the first 100 results were screened to identify keywords and synonyms; abstracts of relevant papers were also screened for relevant vocabulary. This enabled refinement of the search terms which were then adapted to various databases search engines (online supplemental appendix 2).

### Screening and selection of sources of evidence

The resources were downloaded to EndNote V.21^19^ and duplicates were removed. Resources were then uploaded to Rayyan^20^ to conduct two rounds of blinded screening, first titles and abstracts and then full-text screening. Resources matching the inclusion criteria were moved to the next round. The selection process was undertaken by two screeners (NP, AA). Conflicts were resolved by discussion between the two screeners (NP, AA) until consensus was reached; in case of disagreement resources were taken to a third reviewer (AN).

### Data charting process and data items

An extraction sheet was built using Microsoft Excel 2021;^21^ it was constructed based on the conceptual framework as well as through scanning articles likely to be captured in the search and identifying their main features. A pilot was done to ensure the calibration of the extraction sheet with included resources. The pilot revealed the need to use NVivo V.12,^22^ in addition to the extraction sheet, to ensure efficient extraction of included resources. We also conducted a pilot in NVivo using one included resource to ensure consistent data extraction. Data was extracted by one reviewer (NP), and it was checked by two other reviewers (AN, SM).

The research team agreed to extract data on (1) general information about the resource, (2) functions/components/processes/elements of health research systems and (3) information about tools and indicators. The full data extraction framework can be found in online supplemental appendix 3 and the data extraction sheet is available online.^23^ In NVivo, the conceptual framework’s categories served as themes for coding the resources. We also allowed new themes to emerge to ensure we captured all relevant results. The coding book is available online.^24^

### Synthesis of results

We undertook descriptive statistics and further analysis using framework synthesis to present findings for each theme. Realist, semantic, theoretical and inductive thematic analysis was used to analyse the results.^25,26^ We also assessed the relationships between themes at two levels of analysis, using Pearson’s correlation analysis in R (V.4.1.2).^27^ Themes were coded as binary variables, with 1 indicating the presence of a theme and 0 indicating its absence. At the article level, we calculated pairwise correlations to assess the extent to which themes co-occurred across articles. At the coded-extract level, pairwise correlations were calculated to assess the extent to which themes co-occurred within individual coded extracts. By convention, we used thresholds of 0.10 for small correlations, 0.30 for medium ones and 0.50 for large correlations.

## Results

### Selection of sources of evidence

The search was run on the 24 June 2025 and retrieved 5056 resources. Resources were uploaded in EndNote 21 where duplicates were removed, leaving 2682 resources to be screened. Resources were uploaded to Rayyan for the two rounds of blinded screening. After the titles and abstracts screening, 2553 resources were excluded (95%) and 129 resources were included (5%). The remaining resources were screened for full-text, resulting in 93 (70%) excluded and 36 (30%) included for analysis. The flowchart is presented in figure 2.

**Figure 2:**
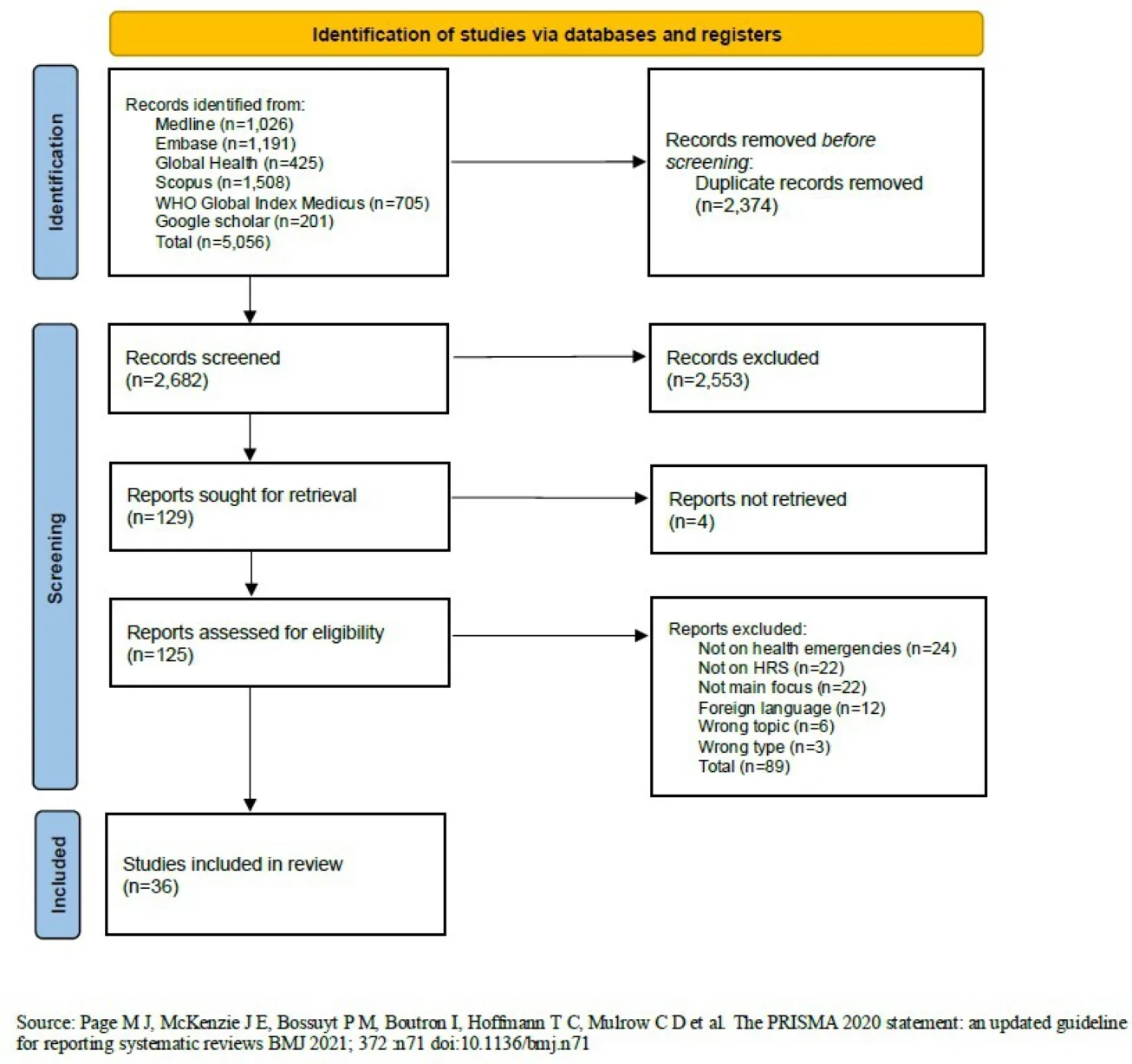
PRISMA flowchart diagram for resources selection. HRS, Health Research System; PRISMA, Preferred Reporting Items for Systematic Reviews and Meta-Analyses.

### Synthesis of results—general summary

All 36 resources were published after 2016, most resources are opinion pieces (22/36, 61%)^1,3,4,28–46^ and the rest are research articles (12/36, 33%)^13,47–57^ or reports (2/36, 6%).^58,59^ The scope of the included resources is mainly global (18/36, 50%),^3,4,13,28,29,31,37–40,43,45,48,52,54–56,58^ with other resources having regional (7/36, 19%),^30,35,42,47,50,53,59^ multinational (5/36, 14%),^1,33,34,36,41^ national (5/36, 14%)^32,46,49,51,57^ or subnational scopes (1/36, 3%).^44^ Regional resources focus on Africa (6/7, 86%)^30,35,42,50,53,59^ and Asia-Pacific regions (1/7, 14%).^47^

While all resources discuss health research systems in one or more aspects, only seven (7/36, 19%)^1,4,13,46,51,52,57^ were using a specific framework for their analysis and only two used the Pang *et al* framework.^1,57^ The coding and mapping of the resources to the functions/components/processes/elements of the conceptual framework showed great discrepancies between mentions of the different characteristics. The ‘build, strengthen and sustain capacity’ component was mentioned in 86% of the resources (31/36)^1,4,13,28–32,34–43,46–53,55–59^ and the component ‘secure and allocate funds effectively’ in 75% (27/36).^1,4,13,28–32,34,35,38–43,46–52,54,56,57,59^ Figure 3 summarises coding across all framework characteristics and emerging themes.

**Figure 3:**
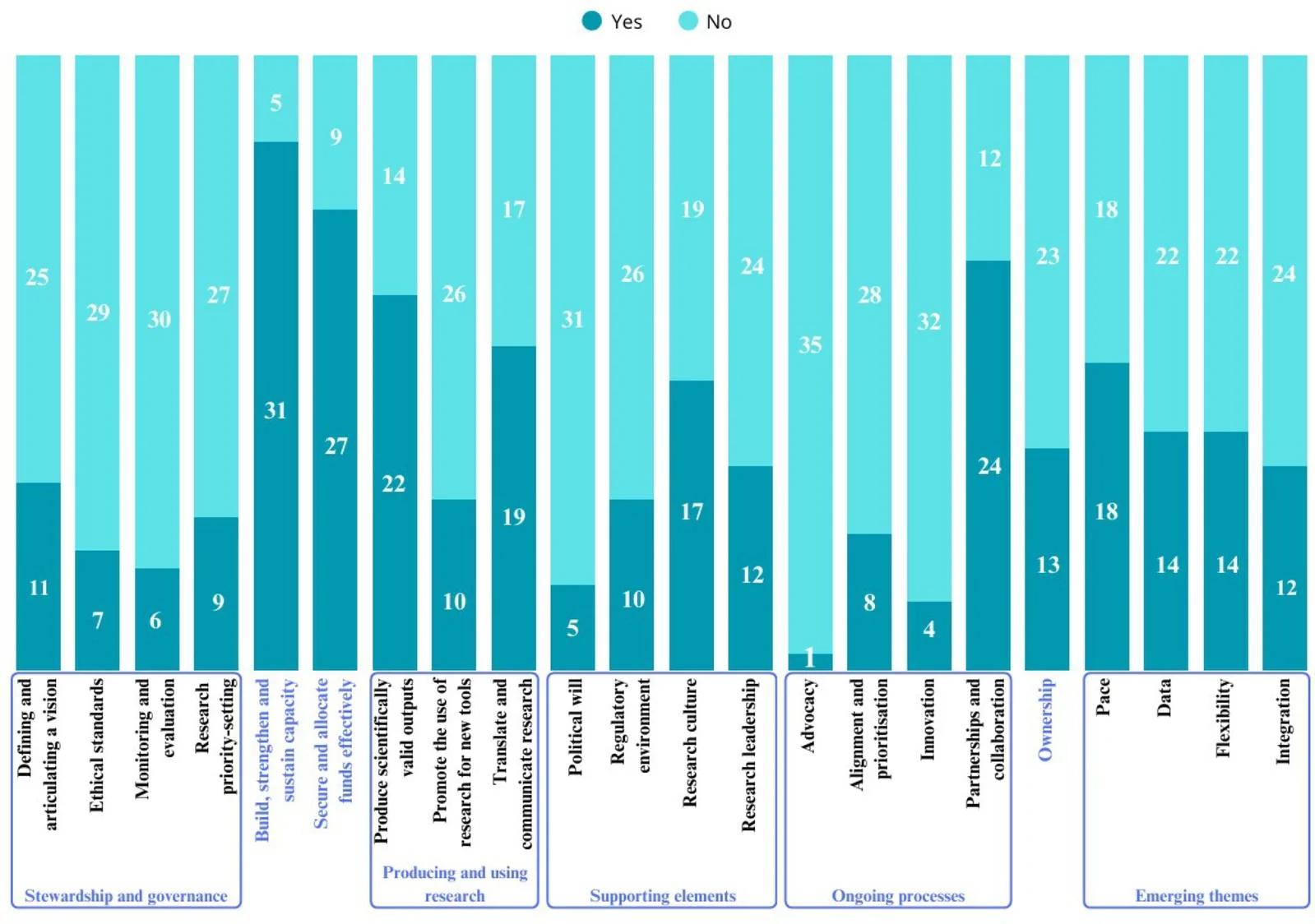
Bar chart representing the coding and mapping of the resources to the Jones framework. Blue text represents groupings according to the various components of the framework.

### Synthesis of results—interconnectedness between characteristics

The characteristics of health research systems seem to display deep and complex relationships, as 192 of 792 (24%) extracts were coded to at least two themes. Three characteristics seem to be strongly related: ‘build, strengthen and sustain capacity’, ‘secure and allocate funds effectively’ and ‘partnerships and collaboration’ (figure 4). Overall, this represents the complexity and interconnectedness of health research systems’ characteristics.

**Figure 4:**
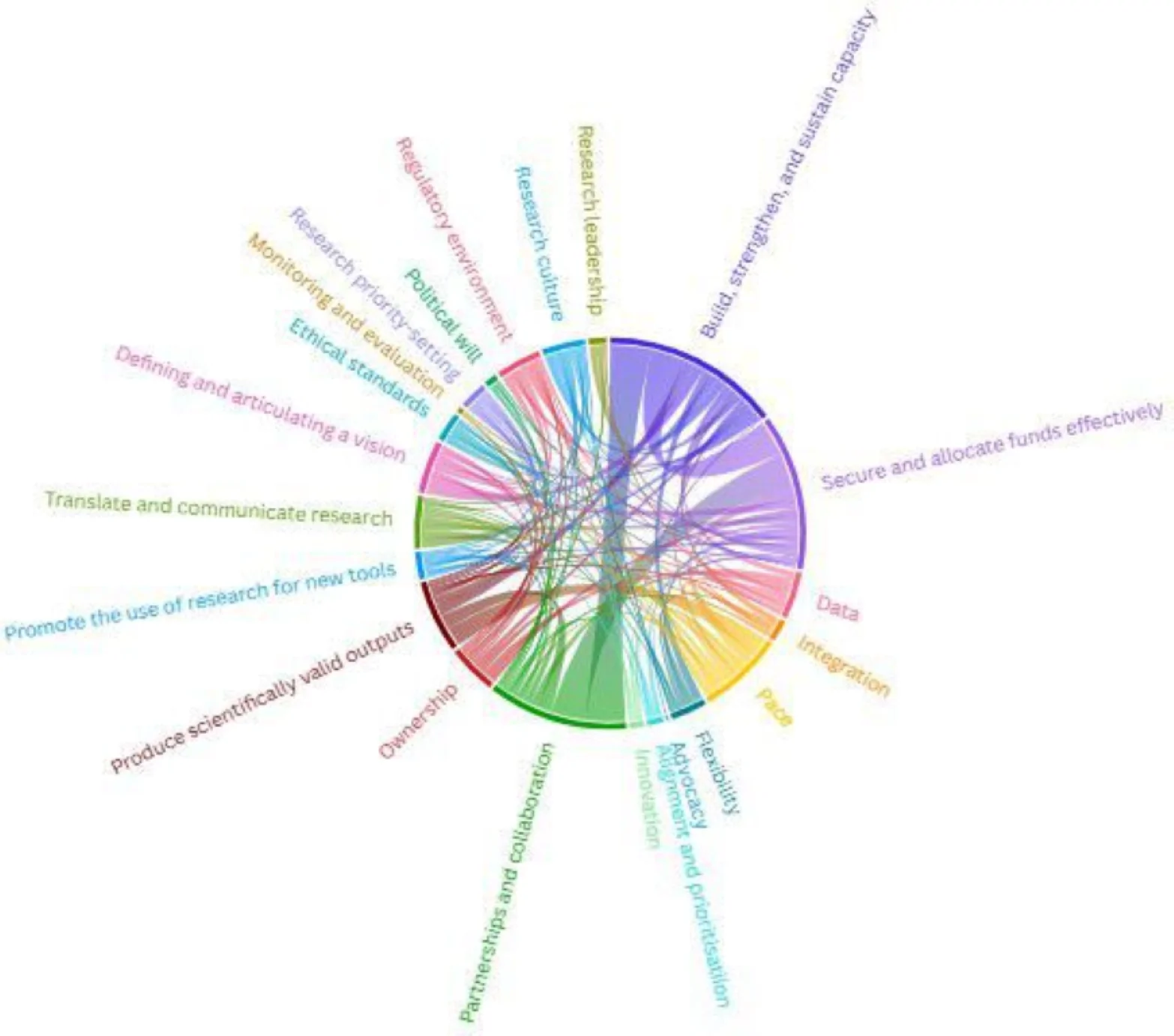
Chord diagram representing the interconnectedness of the various characteristics of health research systems for health emergencies preparedness and responsiveness (those coded to multiple categories only, n=192).

Online supplemental appendix 4 represents the linkages between the different parts of the health research system based on the number of common themes at the coding extract level. The Pearson coefficient estimates the direction and intensity of the correlation between themes. The correlation coefficient ranges from −1 to 1; a score of 0 indicates no linear relationship, a score between −1 and 0 indicates a negative relationship and a score between 0 and 1 indicates a positive relationship. The closer the coefficient gets to −1 and 1, the stronger the relationship. Pearson coefficient analysis at the coded extract level shows only small positive (max c=0.21) or negative correlations (min c=−0.15) or no correlation. The strongest effects were observed between ‘ethical standards’ and ‘regulatory environment’, which showed a small positive degree of association at the extract level; and between ‘partnerships and collaboration’ and ‘translate and communicate research’, which showed a small negative degree of association at the extract level.

Finally, the correlation coefficient at the article level appears more conclusive, suggesting that when one resource mentions one specific theme, it is more or less likely to mention any other given theme (figure 5). For instance, ‘defining and articulating a vision’ and ‘political will’ show a strong positive degree of association at the article level (c=0.61). Some themes also exhibit small negative correlations such as ‘securing and allocating funds effectively’ and ‘translate and communicate research’ (c=−0.29). Some themes show no correlation such as ‘securing and allocating funds effectively’ and ‘ownership’ (c=0.03). The correlation analysis implies that these complex systems cannot be thought of in silos and that any intervention needs careful consideration, as it is likely to impact several characteristics of health research systems. Characteristics which display high correlations might have stronger linkages and impacting one risks impacting the other.

**Figure 5:**
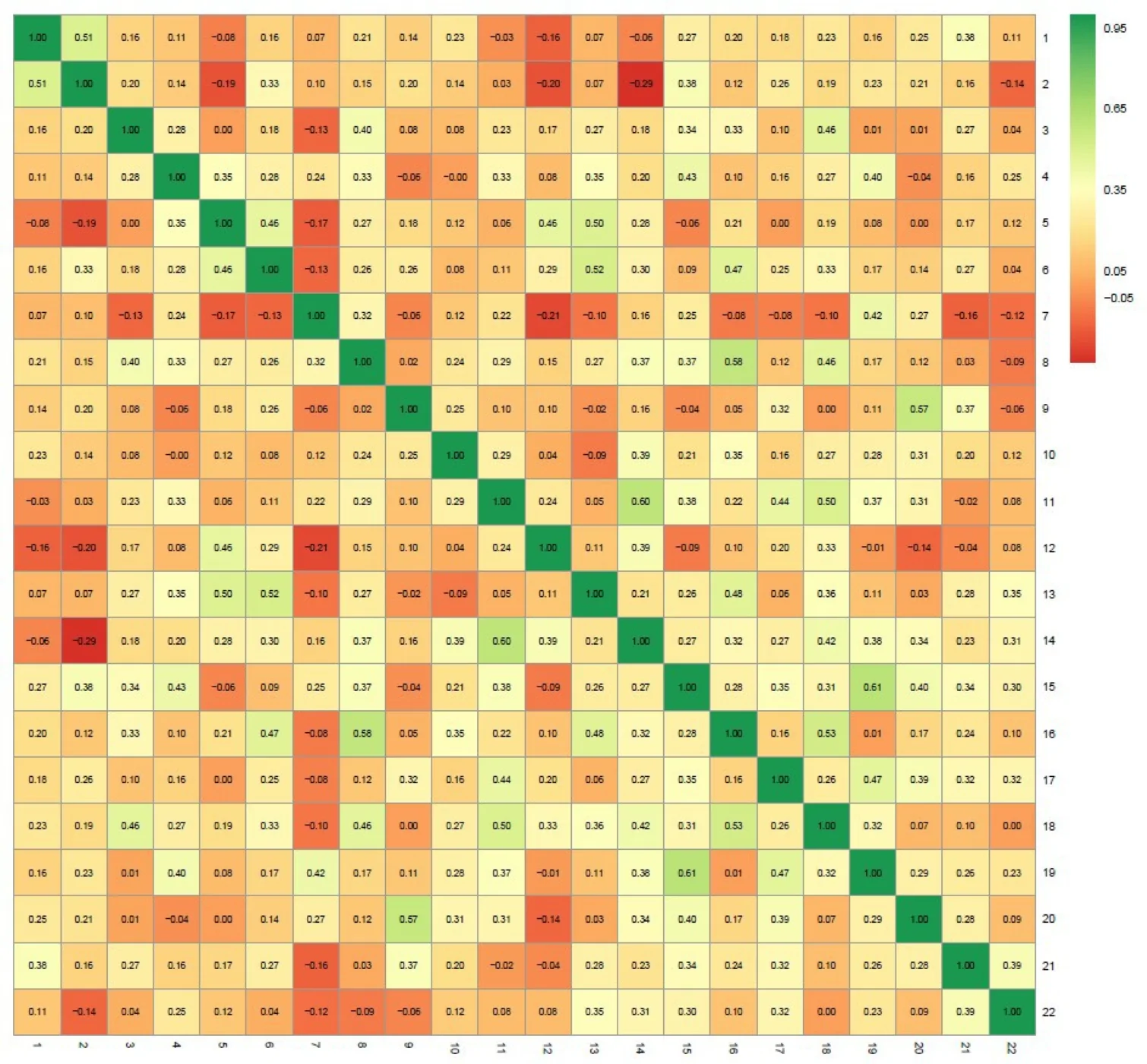
Heatmap showing the Pearson coefficient between coding categories at the article level. If the coefficient is equal to 1 it indicates perfect positive correlation, if it is equal to −1 it indicates perfect negative correlation, the more it tends to 0 the weaker the correlation. Characteristics have been renamed for formatting reasons; the corresponding list can be found in online supplemental appendix 4.

### Synthesis of results—key characteristics

#### Creating and sustaining resources

On the human resources side, there seems to be a lack of local skilled workforce across a range of research themes^32,37,42,46,49,51^ (epidemiology, laboratory, surveillance, ethics) but also regarding ‘core transferable competencies’^32,37,46,55–57^ such as research management.^57,59^ Brain drain is also a major preoccupation for low- and middle-income countries.^32,37,55,56^ In order to enable preparedness and response to health emergencies, there is a need to strengthen local workers and locally-owned institutions’ competencies by increasing research ownership^1,41,49,57^ as well as to enable bidirectional learning between different research environments.^39,57^ At the institutional level, there also seems to be a lack of capacity for response to health emergencies.^32^ Centres of excellence appear to play a key role in enabling capacity strengthening^28,35,42,50,59^ and universities are also flagged as central to provide adequate training.^1,28,41,50,59^ The potential role regional organisations could play to strengthen institutional capacity was also put forward.^30,35,50^ Finally, on the infrastructure aspect, existing resources are deemed insufficient, notably in low- and middle-income countries.^28^ Focus is being put on data infrastructure, which has the potential to provide access and use of data for health emergencies response.^4,13,28,31,41,46,48,57^ Authors also recognised the need for minimum infrastructure capacity to allow for responsiveness.^37,38^ Overall, key enablers of health emergency response are long-term and sustained investments in capacities^1,13,55^ in a transversal manner across health research systems.^4^ Research capacity strengthening efforts would benefit from increased funders and implementation coordination to reduce financial waste and increase return on investments.^1,13,30,32,35,38,46,47,49,50,52,57,58^

#### Secure and allocate funds effectively

There is broad consensus that research funding is insufficient, notably in low- and middle-income countries, even though financial resources are an essential enabler for preparedness and response to health emergencies^4,13,29,31,39,42,46,47,51,56,57,59^ as was shown during the COVID-19 pandemic.^1,40,59^ Major imbalances in research funding between high-income countries and low- and middle-income countries have been highlighted,^28,47^ these may act as a barrier if they hinder ownership, local priorities recognition and capacity development.^30,41,50,52,59^ This aligns with recognition of the need to augment national investment in research systems^1,31,35,48,49,52,59^ and diversify funding sources to increase sustainability.^13,46,52,57^ Funding also suffers from a lack of transparency and poor data availability^1,40,47,49,57^ and authors have called for tools and metrics to better allocate and monitor investments in health research.^28,38,43,52,59^ Again, long-term, sustainable funding across all elements of health research systems and pathogens is an enabler of an efficient response to health emergencies.^13,29,34,35,54,59^ This component of health research systems appears to be a central lever to enable rapid changes in the system, for instance by altering incentives or channelling funding to specific domains.^1,5,13,40,46,57^ Finally, it is highlighted that it is much less expensive to prepare for pandemics than to suffer the multiplied cost of the response.^43^

#### Stewardship and governance

It appears that it is considered the State and governments’ responsibility to be the main enablers of health research systems functioning during health emergencies^1,29,49,51,52,59^; they can do so by enacting adequate regulations and providing sufficient funds.^13,29,46,49,57^ Having a comprehensive health research system and an underlying strategy, such as in the UK or in New Zealand, appears to be a key factor in effective response.^1,13,57^ Regional organisations might also have a role to play in the uniformisation and development of these policies, but this needs to be further developed.^35,50^ Ethics systems are a cornerstone of this governance,^13,37,57^ they need to be adaptive and able to provide quick insights into research projects^1,4,13,39^; they may also support improved local ownership of research by enacting rules on collaboration and data sharing practices.^49,57^ The need to monitor the functioning and the impact of health research systems is central to steer the system and to allocate funds effectively.^1,13,31,35,38,52,59^ The role of regional organisations in this aspect might be quite important to promote harmonisation.^35^ Finally, research priority-setting is also a key element of governance and fund allocation during health emergencies.^1,4,13,46,47,53,57^ Prioritisation processes need to be locally owned and relevant,^4,47,57^ centralised to avoid unnecessary redundancy,^1,13,57^ inclusive to reflect the whole of society^1,15,53,57^ and to consider short- and long-term priorities.^1,53^

#### Producing and using research

Research across all disciplines and varied designs is central to health emergency response because it enables evidence-based decision-making and saving lives.^3,13,31–33,39–41,45,46,48,53,57,58^ However, during the COVID-19 pandemic, there was significant waste resulting from poorly designed trials^1,47^ which could have been improved through enhanced coordination and data sharing.^1,3,13,41,57,58^ One enabler appears to be the flexibility of research design to adapt as pathogens evolve, new drugs are discovered and evidence accumulates.^1,13,33,39,46,57^ Countries also need to have locally relevant research questions addressed with local capacities to produce valid research outputs.^4,36,41,46,56–58^ The ability to promote research and develop new tools is also central to health emergency response as it can improve health outcomes.^1,37,45^ This requires partnerships between research and industries as well as speed and funding.^1,4,56,57^ The roles of implementation research and integration between research and care are central in this regard as this allows the quick deployment of new tools.^13,34,46,56,57^ Finally, the translation and communication of research results is important in mitigating the health and social effects of epidemics and build trust.^13,34,44,46,57^ However, current systems appear inadequate for health emergencies.^1,3,36,37,44,45,59^ A central issue is the handling of misinformation as this hinders effective response.^4,36,37,45^ Another problem is the disconnection between research and policymaking which hampers efficient use of results and timely communication to the general population.^1,3,13,44,46,57^ These challenges can be addressed through the development of infrastructure for knowledge translation,^1,57^ an increase in collaborations between all sectors of society^1,37,46,53,57–59^ and the deployment of implementation research.^13,37,39,46,57^

#### Supporting elements

Political will is an enabler element for health research systems; it allows capacity strengthening, fund allocation and effective governance.^1,35,46,59^ This element can be hindered by reluctant politicians or institutional arrangements.^1^ For regional organisations to play a role in health research systems, there also needs to be a political will to displace sovereignty on this topic.^35,50^ The regulatory environment is also an important element that can support health research systems, without an appropriate environment health research systems can hardly function.^13,29,35,37,43,50,59^ Again, this needs to cover all the characteristics of health research systems from legal preparedness mechanism^13,35^ to countermeasures development^35^ and ethical^4,13,59^ or data sharing regulations.^4^ Research culture and research leadership are two interrelated aspects which can ensure health research systems development and functioning;^1,13,34,37,46^ in countries where these aspects are well-developed and institutionalised, the research system is more efficient.^31^ International partnerships,^35,36,40^ dedicated funds^34,49^ and sustainable jobs, educational paths and infrastructures^4,36,41,49,57,59^ can all help implement research culture and research leadership. Resilience has been deemed an important feature of the research culture to better face shocks and remain efficient in the face of uncertainties.^4,37,41,46,56,57^

#### Ongoing processes

The emphasis was put on partnerships and collaborations as a cornerstone of health emergency preparedness and response, as it allows common research agendas, streamlined funding and avoids financial waste, focused research efforts and faster research outputs.^1,3,13,30,32,37,41,46,48,52,57^ Furthermore, long-term collaborations appear more effective than improvised ones during emergencies.^32,35,50^ This collaborative mindset should apply across all characteristics of health research systems,^1,13^ across all disciplines^1,13,46,57^ and across all geographical scopes^13,49,52^ or institutional^35,39,46,57^ and societal levels.^1,46,57^ However, some setbacks may hinder effective collaboration such as inequity which can reflect in funding schemes or data sharing practices^48,49^ but also in designation of research lead or research outputs.^41^ Financial collaborations might also be at the expense of the funded country which might be dependent on funding streams which may impact on the development of local health research systems.^41,50^ Collaboration must therefore come with safeguards for equality and equity.^3,39,48^ Collaboration can be hindered by a number of factors such as discrepancies in priorities^1^ or lack of political engagement.^41,50^ Innovation is another ongoing process which relies on the research and development ecosystem;^59^ it needs funding and market incentives.^43^ Finally, advocacy is also an important process which regional organisations might be able to support.^50^

#### Ownership

Ownership is at the centre of health research systems and a key aspect of their sustainability and functioning.^13,15,39,41,53,57,59^ It refers to the fact that a country’s health research system and its capacities are determined, governed and oriented towards meeting its own needs; rather than being shaped by the influence of foreign countries or external donors. This ownership can be enabled by reducing financial dependency,^35,50,53,59^ having strong local leadership^39,41,53,59^ and representing vulnerable populations and communities.^13,57^ This has to apply across other domains of the health research systems in order for that representation and integration to be efficient.^13,57^

### Synthesis of results—emerging themes

Four emerging themes appeared to play an important role in health research systems for health emergencies preparedness and response: pace (18/36, 50%), data (14/36, 39%), flexibility (14/36, 39%) and integration (12/36, 33%).

The fast pace refers to the rapidity applied across health research systems’ characteristics; it involves shortening usual timelines in response to an emergency. This can be supported by regulatory and organisational mechanisms to increase pace when needed. A fast pace in health research systems may enable the containment of diseases to limited geographical areas and produce life-saving countermeasures.^1,13,32,48,53^ This accelerated pace is useful for most characteristics of health research systems; it has been identified as applying to funding,^4,13,57^ review boards,^1,13,32,33^ regulatory processes,^1,13^ research activation,^53,59^ research models,^53,59^ publication,^1,3,37,46^ dissemination and implementation.^45,46^ One enabling factor seems to be long-term investment in research infrastructure and culture.^1,46,56,57^ It was cautioned that this pace should still enable transparency and accountability in those processes.^4,13^ Similarly, rapid publication comes with challenges in quality and dissemination which the development of new publication models could account for.^1,13^ This is best described by the following sentence ‘Make a balance between rigorousness and promptness of research’.^4^

Data appear to be a central element of health research systems, across the entire framework. Data refer to the availability, interoperability and transparency of research results as well as epidemiological data or other data relevant to health emergencies. Data can ensure efficient and contextual decision-making as well as support management of health emergencies.^4,13,41,44,46,51,52,57^ Data are needed across a range of areas such as surveillance, management,^41^ social sciences, biomedical sciences.^4,40,44^ In order for data to fulfil its role it needs to be easily available,^1,4,13,38,46,47,49,57^ sustained by necessary infrastructure,^13,38,46,49,51,57^ shared and widely available; finally data also need interoperability and coordination.^4,13,53,57^ To ensure the sharing and use of data, the right incentives must be attached^41^ to these measures and there needs to be a clear regulatory framework.^4^ No specific enablers for data have been mentioned in the resources.

Flexibility and the ability to pivot also seem to be very relevant to health emergencies preparedness and response. It refers to mechanisms that build resilience within health research systems, enabling them to quickly adapt to emergencies. Indeed, this flexibility can support pace and ensure efforts can be rapidly and efficiently diverted from one area to another. Once again, this mechanism seems to be transversal to all characteristics of health research systems^13,37,40,46,54,59^: it is needed at the funding level,^1,4,13,54,57^ knowledge mobilisation and dissemination level,^1,32,54,59^ the research model level,^1,39,40^ the surveillance level,^41^ the infrastructural and human capacity level^32,34,37,40,41,46,56,59^ and at the governance level.^1,13,46^ Enablers for flexibility appear to be adequate capacity-strengthening specifically oriented towards flexibility^40,56^ and a transversal approach to research programmes and strengthening initiatives as opposed to more vertical programmes.^41^

Finally, integration has been mentioned as one core characteristic for health emergencies preparedness and response. Integration refers to the integration between health research and healthcare systems.^1,3,13,34,40,42,46,51,57^ It supposes research and care are closely interrelated, through specific coordination mechanisms. Integration appears to support health emergencies response and most notably pace, discovery^1^ and rapid implementation of research results.^13,34,57^ It also ensures coordination^1,42,50,51^ and avoids funding waste.^42^ Integration can also be applied across various geographical scales of research,^57,59^ research actors and for surveillance matters.^40,42,53,59^ Enablers of this integration appear to be a ‘comprehensive health research strategy’ and long-term investments in research capacities.^1,34,51^

### Synthesis of results—indicators of health research systems

Regarding the secondary objective of this review, to identify indicators specifically designed to assess the preparedness and responsiveness of health research systems to health emergencies, no indicators have been identified. Some resources (11/36, 31%) mentioned or proposed indicators of health research systems outside of health emergencies, such as mapping research funding flows^47^ or tracking the number of publications with an author affiliation in a given country.^52^ However, none of the identified indicators was specific to health emergencies. Some resources acknowledged the usefulness of indicators for funding decisions.^1,38,51^

## Discussion

This study, to our knowledge, is the first scoping review to systematically map the desired characteristics of health research systems to address health emergencies. The conceptual framework analysis has shown that all characteristics of health research systems appear to play a role in health emergency preparedness and response as confirmed in the recent literature.^14^ Some characteristics such as ‘creating and sustaining resources’, ‘securing and allocating funds effectively’, as well as ‘partnerships and collaborations’ appear to be especially critical. Another important finding is the interconnectedness between all characteristics of health research systems but also between these three central tenets. Interestingly, some conceptually related characteristics show negative relationships. For example, ‘collaborations and partnerships’ and ‘translate and communicate research’ are negatively linked, despite collaborations being central to implementing research results. This suggests these connections may be less direct than expected and highlights the need for further research and recognition of these essential linkages. These findings outline the need for health research systems strengthening initiatives to adopt a system approach and consider carefully the linkages between the various elements of the system.^14^ Future investments in health research systems should focus on horizontal, sustained funding to ensure their effective functioning. There is an important interconnection between some of the areas of health research systems which shows that all are intrinsically linked and that potential actions will require multifaceted interventions.

Health research systems require all framework characteristics to function during health emergencies, but four themes appear to play a specific role for health emergency preparedness and response: integration, pace, data and flexibility. Pace and flexibility appear to be necessary operating modes during emergencies, while data and integration are also needed outside of health emergencies. Efforts should be directed to strengthening mechanisms to sustain pace and flexibility during peacetime. Data and integration can be reinforced alongside other characteristics of health research systems and also need sustained investments. It would be important to investigate the nature of health research systems’ characteristics since some appear to play a structural role, while some refer to functional elements of the system. Furthermore, the conceptual framework does not capture the deployment of health research systems during health emergencies and how it contributes to containing threats.

This study is the first to focus on all income levels, whereas all similar previous studies had a focus on high-income or higher middle-income countries.^1,4,13,14^ The body of resources included varied in geographical scope, from subnational to global, which made it difficult to identify discrepancies across income levels. However, there does not seem to be a major difference in how health research systems need to function during health emergencies. The difference lies in the current infrastructural and human capacities, which appear from our data to be lower in low- and middle-income countries and need further strengthening. Another key difference is that of domestic funding mobilisation which is a pervasive issue in low-income settings. While health research systems are mainly studied through the national lens, during health emergencies, some research needs align across the world, and findings from one context might be used in another. This is captured by the ‘producing and using research’ function which still suffers from deficiencies.

Finally, no indicators with a focus on preparedness and response of health research systems to health emergencies have been identified. All indicators identified related to health research systems in general and were loosely defined. This might suggest that the conceptualisation of health research systems within health emergencies is a recent preoccupation. This might also relate to the fact that a functioning health research system outside of emergencies might be able to respond effectively to health emergencies; and therefore, a monitoring and evaluation framework relevant to health research systems in general would be sufficient. Developing further indicators for pace and flexibility, which are two important features of health research systems during emergencies, would be important.

This study also has limitations. First, the conceptual framework used was not made specifically for a health emergency scope. Therefore, the thematic analysis allowed for the identification of four new themes which may enable to start envisioning what health research systems should look like in order to be prepared and responsive to health emergencies. Second, only one reviewer conducted the data extraction; therefore, as mentioned in the methods, we undertook a pilot, checked by two other reviewers, to mitigate this issue. Third, the objective of identifying and mapping indicators has only been partially achieved due to the lack of literature identified on this topic. Fourth, as is usual for scoping reviews, the quality of the resources was not appraised, however we presented general information on the included resources such as their type. However, it is interesting to note the high proportion of opinion pieces (61%) among the included resources, which indicates the relatively weak evidence base and the need for further studies on this topic. Finally, the choices to exclude non-English resources, to limit the screening to the first 10 pages of Google Scholar and to rely on the WHO list of high-consequence infectious diseases for the selection of resources might result in missed documentation, but were guided by practical considerations.

## Conclusion

Health research systems are central to support and maintain the health status of populations. During health emergencies, these systems suffer from high pressure along with alterations in their functioning. This scoping review highlighted the characteristics and elements of health research systems that play an essential role during the response to health emergencies, emphasising ways to improve their preparedness. Four new characteristics have been outlined and appear to play a central role in response to health emergencies. Given the current atmosphere of funding contraction and undermining of health research systems, it is essential to show how these systems contribute to improving health and protecting populations and advocate for sustained investment in the strengthening of health research systems, notably towards health emergencies preparedness and response. Future investments in health research systems should consider the interconnectedness of these complex systems and take a horizontal approach. Furthermore, policymakers and research capacity funders should invest continually and sustainably in all characteristics of health research systems if they want to support health emergency preparedness and response. Finally, there is a need to develop a monitoring framework to evaluate the capacity of health research systems to respond to health emergencies.

## Supplementary material

10.1136/bmjph-2025-004023 online supplemental file 1

10.1136/bmjph-2025-004023 online supplemental file 2

10.1136/bmjph-2025-004023 online supplemental file 3

10.1136/bmjph-2025-004023 online supplemental file 4

## Data Availability

Data are available in a public, open access repository.

## References

[1] Hanney SR, Straus SE, Holmes BJ (2022). Saving millions of lives but some resources squandered: emerging lessons from health research system pandemic achievements and challenges. Health Res Policy Syst.

[2] Lurie N, Keusch GT, Dzau VJ (2021). Urgent lessons from COVID 19: why the world needs a standing, coordinated system and sustainable financing for global research and development. Lancet.

[3] Turner T, El-Jardali F (2020). The crucible of COVID-19: what the pandemic is teaching us about health research systems. Health Res Policy Syst.

[4] Yazdizadeh B, Majdzadeh R, Ahmadi A (2020). Health research system resilience: lesson learned from the COVID-19 crisis. Health Res Policy Syst.

[5] Pang T, Sadana R, Hanney S (2003). Knowledge for better health: a conceptual framework and foundation for health research systems. Bull World Health Organ.

[6] Jones CM, Ankotche A, Canner E (2021). Strengthening National Health Research Systems in Africa: Lessons and Insights from across the Continent.

[7] Antonio E, Pulik N, Ibrahim SK (2025). Research prioritisation in preparedness for and response to outbreaks of high-consequence pathogens: a scoping review. BMC Med.

[8] World Health Organisation (2005). International health regulations.

[9] World Health Organisation (2024). International health regulations.

[10] World Health Organisation Global research and innovation for health emergencies: building the world’s resilience against future outbreaks and pandemics. 2023.

[11] Coalition for epidemic preparedness innovations. why we exist?. https://cepi.net/about/whyweexist/%20(2024.

[12] World Health Organisation (2025). Proposal for the WHO Pandemic Agreement.

[13] Embrett M, Carson A, Sim M (2025). Building Resilient and Responsive Health Research Systems:Responses and the Lessons Learned from the COVID-19 Pandemic. Health Res Policy Syst.

[14] Hanney SR, Yazdizadeh B (2025). Health research versus the virus: strengthening systems, saving lives. Health Res Policy Sys.

[15] Peters MDJ, Marnie C, Tricco AC (2020). Updated methodological guidance for the conduct of scoping reviews. *JBI Evid Synth*.

[16] Arksey H, O’Malley L (2005). Scoping studies: towards a methodological framework. Int J Soc Res Methodol.

[17] Tricco AC, Lillie E, Zarin W (2018). PRISMA Extension for Scoping Reviews (PRISMA-ScR): Checklist and Explanation. Ann Intern Med.

[18] Pulik N, Molyneux S, Norton A (2024). Key elements of health research systems for pandemic prevention, preparedness, and response: a scoping review. Open Science Framework.

[19] (2013). The Endnote Team.

[20] Ouzzani M, Hammady H, Fedorowicz Z (2016). Rayyan-a web and mobile app for systematic reviews. Syst Rev.

[21] Microsoft Corporation (2018). Microsoft excel.

[22] Lumivero (2020). Nvivo.

[23] Pulik N, Adenipekun A, Molyneux S (2025). Data Extraction File, Key Characteristics of Health Research Systems for Health Emergencies Preparedness and Response: A Scoping Review.

[24] Pulik N, Adenipekun A, Molyneux S (2025). Coding book and analysis, Key Characteristics of Health Research Systems for Health Emergencies Preparedness and Response: A Scoping Review.

[25] Braun V, Clarke V (2006). Using thematic analysis in psychology. Qual Res Psychol.

[26] Braun V, Clarke V (2021). Thematic Analysis: A Pratical Guide.

[27] R Core Team R foundation for statistical computing.

[28] Adam T, Ralaidovy AH, Ross AL (2023). Tracking global resources and capacity for health research: time to reassess strategies and investment decisions. Health Res Policy Syst.

[29] Boggio A (2020). Human rights and global health emergencies preparedness. J Glob Health.

[30] Carmody P (2023). The Geopolitics of Health Science Research Comment on “The Roles of Regional Organisations in Strengthening Health Research Systems in Africa: Activities, Gaps, and Future Perspectives”. Int J Health Policy Manag.

[31] Cash-Gibson L, Pericàs JM, Martinez-Herrera E (2021). Health Inequalities in the Time of COVID-19: The Globally Reinforcing Need to Strengthen Health Inequalities Research Capacities. Int J Health Serv.

[32] Doumbia S, Sow Y, Diakite M (2020). Coordinating the research response to COVID-19: Mali’s approach. *Health Res Policy Sys*.

[33] Erwin K, Basapur S, Chehab L (2022). Scale, Scope, Speed: Reflections on a Multi-site Covid-19 Study. *She Ji: The Journal of Design, Economics, and Innovation*.

[34] Hanney SR (2023). If Providing Best Care Means Being at the Cutting Edge of Research, Should It Be Implemented System-wide? Comment on “‘We’re Not Providing the Best Care If We Are Not on the Cutting Edge of Research’: A Research Impact Evaluation at a Regional Australian Hospital and Health Service”. *Int J Health Policy Manag*.

[35] Herrero MB, de Oliveira BNL (2023). Health Science Research at a Regional Level: Insights From South America Comment on “The Roles of Regional Organisations in Strengthening Health Research Systems in Africa: Activities, Gaps, and Future Perspectives”. *Int J Health Policy Manag*.

[36] Holmes BJ, MacKenzie A, McManus B (2021). The Covid-19 Pandemic Has Exposed Opportunities To Strengthen Our Health Research Systems. *Clinical and Investigative Medicine*.

[37] Kilmarx PH, Glass RI (2021). Building global health research capacity to address research imperatives following the COVID-19 pandemic. PLoS Med.

[38] Kilmarx PH, Maitin T, Adam T (2020). A Mechanism for Reviewing Investments in Health Research Capacity Strengthening in Low- and Middle-Income Countries. Ann Glob Health.

[39] Kohrt BA, Mistry AS, Anand N (2019). Health research in humanitarian crises: an urgent global imperative. BMJ Glob Health.

[40] Lang T (2022). Enabling evidence to tackle everyday diseases to mitigate another pandemic. Gates Open Res.

[41] Nsanzimana S, Rawat A, Wilson LA (2022). Toward a New Paradigm of North–South and South–South Partnerships for Pandemic Preparedness: Lessons Learned from COVID-19 and Other Outbreaks. Am J Trop Med Hyg.

[42] Onyebujoh PC, Thirumala AK, Ndihokubwayo JB (2016). Integrating laboratory networks, surveillance systems and public health institutes in Africa. Afr J Lab Med.

[43] Saxena A, Baker BK, Banda A (2023). Pandemic preparedness and response: beyond the Access to COVID-19 Tools Accelerator. BMJ Glob Health.

[44] Smits P, Cambourieu C, Ouimet M (2023). Connections between health research systems and decision-making spaces: lessons from the COVID-19 pandemic in the province of Québec, Canada. *Health Res Policy Sys*.

[45] Yousefi Nooraie R, Shelton RC, Fiscella K (2021). Correction to: The pragmatic, rapid, and iterative dissemination and implementation (PRIDI) cycle: adapting to the dynamic nature of public health emergencies (and beyond). Health Res Policy Syst.

[46] Scheithauer S, Hoffmann J, Lang C (2025). Pandemic Preparedness - A Proposal for a Research Infrastructure and its Functionalities for a Resilient Health Research System. Gesundheitswesen.

[47] Antonio E, Pulik N, Lee J (2023). Mapping regional funding for COVID-19 research in the Asia-Pacific region. BMJ Glob Health.

[48] Benach J, Cash-Gibson L, Rojas-Gualdrón DF (2022). Inequalities in COVID-19 inequalities research: Who had the capacity to respond?. PLoS ONE.

[49] Canario Guzmán JA, Espinal R, Báez J (2017). Ethical challenges for international collaborative research partnerships in the context of the Zika outbreak in the Dominican Republic: a qualitative case study. *Health Res Policy Sys*.

[50] Jones CM, Sobngwi-Tambekou J, Mijumbi RM (2022). The Roles of Regional Organisations in Strengthening Health Research Systems in Africa: Activities, Gaps, and Future Perspectives. Int J Health Policy Manag.

[51] Kharazmi E, Ostovar S, Ahmadi Marzaleh M (2023). The need to reorganize health research systems in pandemic crisis: A prospective study. Health Sci Rep.

[52] Kilmarx PH, Maitin T, Adam T (2023). Increasing Effectiveness and Equity in Strengthening Health Research Capacity Using Data and Metrics: Recent Advances of the ESSENCE Mechanism. Ann Glob Health.

[53] Ryan J, Wiyeh A, Karamagi H (2020). A scoping review on research agendas to enhance prevention of epidemics and pandemics in Africa. Pan Afr Med J.

[54] Yaqub O, Luna JA, Moore DA (2022). Responding to a disease with resources from other diseases: Evidence from Zika vaccine research dynamics. Science and Public Policy.

[55] Zachariah R, Dar Berger S, Thekkur P (2020). Investing in Operational Research Capacity Building for Front-Line Health Workers Strengthens Countries’ Resilience to Tackling the COVID-19 Pandemic. Trop Med Infect Dis.

[56] Zachariah R, Maher D, Aseffa A (2020). Strengthening the core health research capacity of national health systems helps build country resilience to epidemics: a cross-sectional survey. F1000Res.

[57] Straus SE, Beckett R, Fahim C (2024). Royal Society of Canada working group on health research system recovery: strengthening Canada’s health research system after the COVID-19 pandemic. *FACETS*.

[58] Rao CY, Goryoka GW, Henao OL (2017). Global Disease Detection—Achievements in Applied Public Health Research, Capacity Building, and Public Health Diplomacy, 2001–2016. Emerg Infect Dis.

[59] Omange RW, Ocholla AO, Kwallah AO (2017). MEETING REPORT: UNESCO-MERCK AFRICA RESEARCH SUMMIT 2015- ACCELERATING ACCESS AND SUSTAINING INNOVATION “FROM AFRICA FOR AFRICA”. Afr J Infect Dis.

