# Supplementary material for: Key characteristics of health research systems for health emergencies preparedness and response: a scoping review
Source: BMJ Public Health. 2026 Nov 2;4(4):e004023. doi: 10.1136/bmjph-2025-004023 (PMC13735364; doi:10.1136/bmjph-2025-004023)
Supplement: Supplementary file 1 [file bmjph-4-4-s001.pdf]

Appendix 1: List of high-consequence infectious diseases.

**Pathogens**	**Global**	**Europe**		**Asia-Pacific**
	**WHO**	**EMERGE**	**ECDC**	**ASEAN**
COVID-19 (SARS-CoV2)	X		X	X
Crimean-Congo haemorrhagic fever virus	X	X		
Ebola virus	X	X		
Marburg virus disease	X			
Lassa fever virus	X	X		
Middle East respiratory syndrome coronavirus	X			
Severe Acute Respiratory Syndrome (SARS)	X			
Nipah and henipaviral diseases	X	X		X
Rift Valley fever	X			
ZikaVirus	X			
"Disease X"	X			
Smallpox		X		
Hendra Virus		X		
Swine Influenza A (H3N2)			X	
Highly pathogenic avian influenza A (H5N1)				X
Low pathogenic avian influenza H7N9				X
Pandemic influenza A (H1N1) 2009 virus				X
Polio virus			X	
Monkey Pox			X	
West Nile Virus			X	
Brucellosis (*Brucella bacteria*)		X		
Q Fever (*coxiella burneti*)		X		
Melioidosis (*Burkholderia pseudomallei*)		X		
Tularaemia (*Francisella tularensis*)		X		
Cholera (*Vibrio cholera*)		X		
Plague (*Yersinia pestis*)		X		
Glanders (*Burkholderia mallei*)		X		
Anthrax (*Bacillus anthracis*)		X		

Table 2: High consequence pathogens to be included in the scoping review and corresponding source organisations by which they were identified (reproduced from Antonio et al., 2023).
