# Supplementary material for: Key characteristics of health research systems for health emergencies preparedness and response: a scoping review
Source: BMJ Public Health. 2026 Nov 2;4(4):e004023. doi: 10.1136/bmjph-2025-004023 (PMC13735364; doi:10.1136/bmjph-2025-004023)
Supplement: Supplementary file 2 [file bmjph-4-4-s002.pdf]

Appendix 2: Search strategy.

"health research system*".mp.
NHRS.mp.
("health research" adj3 capacit*).mp.
("health research" adj3 capabilit*).mp.
("health research" adj3 system*).mp.
("research capacit*" adj3 health).mp.
("research capabilit*" adj3 health).mp.

Box 1: Search strategy for Medline, Embase and the Global Health database (OVID).

"health research system*" OR nhrs OR ("health research" w/3 capacit*) OR "("health research" w/3 capabilit*) OR ("health research" w/3 system*) OR ("research capacit*" w/3 health) OR ("research capabilit*" w/3 health)

Box 2: Search strategy for Scopus

"health research system*" OR NHRS OR ("health research" AND capacit*) OR "("health research" AND capabilit*) OR ("health research" and system*) OR ("research capacit*" and health) OR ("research capabilit*" and health)

Box 3: Search strategy for WHO Global Index Medicus

"health research system*"|NHRS|"health research" AND capacit*|"health research" AND capabilit*|"health research" AND system*|"research capacit*" AND health|"research capabilit*" AND health

Box 4: Search strategy for Google Scholar
