# Supplementary material for: Key characteristics of health research systems for health emergencies preparedness and response: a scoping review
Source: BMJ Public Health. 2026 Nov 2;4(4):e004023. doi: 10.1136/bmjph-2025-004023 (PMC13735364; doi:10.1136/bmjph-2025-004023)
Supplement: Supplementary file 3 [file bmjph-4-4-s003.pdf]

Appendix 3: Data extraction framework.

Characteristics of HRS research
Title
Type of article
Author(s)
Affiliation
Year of publication
Scope of study
Region studied
Country(ies) studied
Income-level of countries studied
Framework used
Discipline/focus
Potential as a case study
**Identifying main HRS elements for health emergencies preparedness and response**
Stewardship and governance
Defining and articulating a vision
Research priority-setting
Ethical standards
Monitoring and evaluation
Financing
Secure and allocated funds effectively
Creating and sustaining resources
Build, strengthen, and sustain the human and physical capacity to conduct, absorb, and utilize health research
Producing and using research
Produce scientfically valid outputs
Translate and communicate research to inform health policy, strategies, practices, and public opinion
Promote the use of research to develop new tools to improve health
Ongoing processes
Advocacy
Alignement and prioritisation
Innovation
Partnerships and collaboration
Supporting elements
Political will
Regulatory environment
Research culture
Research leadership
Inductive coding
**Assessing HRS effectiveness and efficiency with tools and indicators**
Presence or absence of tool
