# Supplementary material for: Key characteristics of health research systems for health emergencies preparedness and response: a scoping review
Source: BMJ Public Health. 2026 Nov 2;4(4):e004023. doi: 10.1136/bmjph-2025-004023 (PMC13735364; doi:10.1136/bmjph-2025-004023)
Supplement: Supplementary file 4 [file bmjph-4-4-s004.pdf]

Appendix 4: Pearson coefficient between coding categories at the extract level.

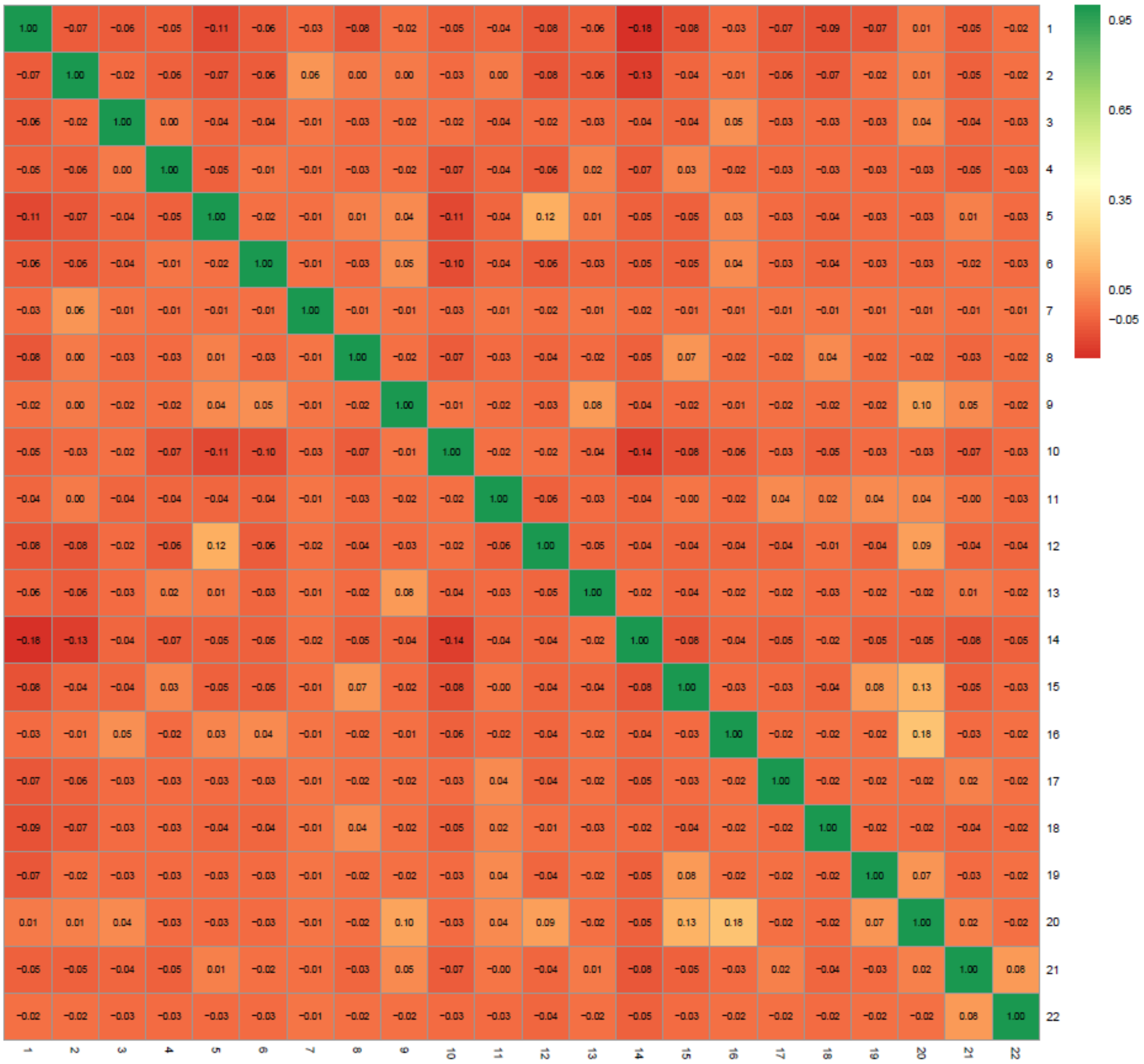

Heatmap showing the Pearson coefficient between coding categories at the extract level. If the coefficient is equal to 1 it indicates perfect positive correlation, if it is equal to -1 it indicates perfect negative correlation, the more it tends to 0 the weaker the correlation.

List of correspondence between numbers and categories:

1	Creating and sustaining resources
2	Secure and allocate funds effectively
3	Data
4	Integration
5	Pace
6	Flexibility
7	Advocacy
8	Alignment and prioritisation
9	Innovation
10	Partnerships and collaboration

11	Ownership
12	Produce scientifically valid outputs
13	Promote the use of research for new tools
14	Translate and communicate research
15	Defining and articulating a vision
16	Ethical standards
17	Monitoring and evaluation
18	Research priority-setting
19	Political will
20	Regulatory environment
21	Research culture
22	Research leadership
